# Potential yellow fever epidemics in unexposed populations

**DOI:** 10.2471/BLT.18.213298

**Published:** 2018-05-01

**Authors:** Duane J Gubler

**Affiliations:** a Duke-NUS Medical School, 8 College Road, Singapore 169857, Singapore.

The global trends of population growth, urbanization and globalization, combined with inadequate public health infrastructure and complacency, have put the world at high risk for infectious disease outbreaks.^1^^–^^3^ Recent unexpected epidemics of diseases that were once controlled or considered unimportant, such as the plague in India (1994), West Nile virus (1999), dengue fever, chikungunya, Ebola (2013–2016) and Zika (2015–2016), as well as newly recognized diseases, such as avian influenza H5N1 (1997), Nipah virus (1998–99), severe acute respiratory syndrome (2003) and influenza H1N1 (2009), underscore this increased risk.^1^^–^^6^ Currently, yellow fever, another disease that has been well-controlled for decades, represents a substantial risk to public health and economic security.^7^

In humans, yellow fever virus causes haemorrhagic disease, with an estimated case fatality rate of 20–50% in patients with severe disease.^8^ Like chikungunya, dengue and Zika viruses, yellow fever is maintained in a sylvatic cycle involving monkeys in rainforests. All of these viruses are also transmitted in an urban cycle comprising humans and *Aedes aegypti* mosquitoes.^1^^,^^5^^,^^8^

Urban epidemics of yellow fever were effectively controlled in the 1950s and 1960s by mosquito control and human vaccination programmes combined with strict enforcement of the International Health Regulations (IHR) requirement that travellers to and from endemic areas show evidence of yellow fever vaccination.^8^ The success of these programmes led to complacency and redirection of resources, which resulted in decreased compliance with vaccination requirements in endemic countries. This situation, combined with less strict enforcement of IHR for travellers, contributed to increasing epidemic transmission in some African and American countries.

Human cases of yellow fever in the past three years are at the highest level in decades, particularly in South America, where there were 3481 reported cases for the period July 2017 – February 2018, more than double the total from the same period the year before. In 2016, 11 workers returning to China from Angola were diagnosed with yellow fever. Fortunately, no known secondary transmission occurred. This was the first time yellow fever had been reported from the Western Pacific and South-East Asian Regions, where approximately 2 billion people live in areas inhabited by *Ae. aegypti*.^9^ The people in these regions have had no previous exposure to yellow fever virus, are not covered by routine vaccination for the disease, and therefore have no immunity.

In this issue of the *Bulletin*, Shannon E Brent et al. estimated the number of air travellers to and from yellow fever virus-endemic countries.^7^ Although these estimates are likely to be conservative,^7^ the data are alarming. With millions of unvaccinated travellers visiting yellow fever virus-endemic countries, it is only a matter of time before people infected with yellow fever virus travel to countries in South-East Asia and the Western Pacific and secondary transmission occurs. The number of unvaccinated travellers who become infected and export the virus between endemic countries is the highest in decades, with 10 cases already confirmed in 2018.^10^ If yellow fever is introduced to their countries, governments are likely to demand vaccine, but the global manufacturing capacity is inadequate. Although the World Health Organization (WHO) estimates that 50 million doses will be produced in 2018, this is not enough to provide emergency vaccination for a major epidemic. A yellow fever epidemic is likely to affect the global air transport system, causing delays and/or shortages in delivery of food and medicines, with potentially significant negative health and economic impact.^11^ Passive laboratory-based surveillance, as currently practiced in most at-risk countries, is unlikely to detect the introduction of yellow fever virus in time to contain its spread. All governments in countries at risk of yellow fever introduction need to adjust their policies and funding priorities accordingly, to ensure adequate surveillance, vaccination and capacity for containment.

WHO’s Eliminate yellow fever epidemics (EYE) strategy calls on countries to improve IHR adherence, strengthen surveillance by improving laboratory capacity and develop more effective urban mosquito control programmes.^12^ The strategy also suggests that endemic countries expand their national immunization programmes to include yellow fever vaccine. However, such expansion will not be feasible until the vaccine shortage is solved. Considering the current high risk of yellow fever spread, a global fund for yellow fever and other *Aedes*-transmitted diseases should be created to fund research, vaccination and capacity building programmes.
